# Genome-Wide Constitutively Expressed Gene Analysis and New Reference Gene Selection Based on Transcriptome Data: A Case Study from Poplar/Canker Disease Interaction

**DOI:** 10.3389/fpls.2017.01876

**Published:** 2017-10-31

**Authors:** Jiaping Zhao, Fan Yang, Jinxia Feng, Yanli Wang, Barbara Lachenbruch, Jiange Wang, Xianchong Wan

**Affiliations:** ^1^State Key Laboratory of Tree Genetics and Breeding, Institute of New Forestry Technology, Chinese Academy of Forestry, Beijing, China; ^2^Department of Forestry, College of Forestry, Jiangxi Agricultural University, Nanchang, China; ^3^Department of Horticulture, School of Horticulture Landscape Architecture, Henan Institute of Science and Technology, Xinxiang, China; ^4^Department of Forest Ecosystems and Society, Oregon State University, Corvallis, OR, United States

**Keywords:** high-throughput sequencing, expression stability, house-keeping gene, internal control, differential expression, integrate analysis, poplar, *Botryosphaeria dothidea*

## Abstract

A number of transcriptome datasets for differential expression (DE) genes have been widely used for understanding organismal biology, but these datasets also contain untapped information that can be used to develop more precise analytical tools. With the use of transcriptome data generated from poplar/canker disease interaction system, we describe a methodology to identify candidate reference genes from high-throughput sequencing data. This methodology will improve the accuracy of RT-qPCR and will lead to better standards for the normalization of expression data. Expression stability analysis from xylem and phloem of *Populus bejingensis* inoculated with the fungal canker pathogen *Botryosphaeria dothidea* revealed that 729 poplar transcripts (1.11%) were stably expressed, at a threshold level of coefficient of variance (CV) of FPKM < 20% and maximum fold change (MFC) of FPKM < 2.0. Expression stability and bioinformatics analysis suggested that commonly used house-keeping (HK) genes were not the most appropriate internal controls: 70 of the 72 commonly used HK genes were not stably expressed, 45 of the 72 produced multiple isoform transcripts, and some of their reported primers produced unspecific amplicons in PCR amplification. RT-qPCR analysis to compare and evaluate the expression stability of 10 commonly used poplar HK genes and 20 of the 729 newly-identified stably expressed transcripts showed that some of the newly-identified genes (such as SSU_S8e, LSU_L5e, and 20S_PSU) had higher stability ranking than most of commonly used HK genes. Based on these results, we recommend a pipeline for deriving reference genes from transcriptome data. An appropriate candidate gene should have a unique transcript, constitutive expression, CV value of expression < 20% (or possibly 30%) and MFC value of expression <2, and an expression level of 50–1,000 units. Lastly, when four of the newly identified HK genes were used in the normalization of expression data for 20 differential expressed genes, expression analysis gave similar values to Cufflinks output. The methods described here provide an alternative pathway for the normalization of transcriptome data, a process that is essential for integrating analyses of transcriptome data across environments, laboratories, sequencing platforms, and species.

## Introduction

With the development of new generation high-throughput sequencing technology, terabytes (TB) or more of sequencing data are being generated daily from different species, tissues, cells, or environments. These data are then uploaded to public platforms, research centers and laboratories. Most of these sequencing data are derived from RNA based sequencing techniques, such as RNA-seq, microRNA sequencing, or long-noncoding RNA (lncRNA) sequencing. The focus of much research is on differential expression (DE) analysis, which makes use of detailed simultaneous expression information from tens of thousands of genes from different species, tissues, and cells, and also information on cells in different environments or developmental stages. DE analysis gives insight into gene/function relationships. Beyond DE genes, however, are the thousands of genes that are constitutively expressed (CE) in various cells or stages, regardless of environmental conditions. For example, 22.7% of the rice transcripts are CE in 39 different rice tissues (Wang et al., 2010). The ubiquitin extension protein (uep1) gene was identified as a CE gene in oil palm (Masura et al., 2010) and the gene encoding the RNA polymerase II large subunit cyclobutane pyrimidine dimmers was CE in *Arabidopsis thaliana* (Fidantsef and Britt, 2011). The maize brown midrib2 (bm2) gene encodes a methylenetetrahydrofolate reductase that contributes to lignin accumulation (Tang et al., 2014). HK genes are constitutive genes that are transcribed at a relatively constant level in different cells, stages or environments. They are implicated in many basic cellular processes and functions, such as protein synthesis and regulation, translation, and transcription. As such, HK genes are useful reference points for comparative gene expression analysis (Czechowski et al., 2005); Actin, EF1α, tubulin, UBQ, 5.8S rRNA, and 18S rRNA are examples of HK genes commonly used to normalize gene expression data.

However, the assumption of relatively constant expression may not be supported in all cases. Some studies show that that no single HK gene is expressed stably in every environment, and the commonly used HK genes are only expressed consistently in specific cell types or under specific experimental conditions (Thellin et al., 1999; Vandesompele et al., 2002; Andersen et al., 2004). Because of these results, it is increasingly recognized that an important step in gene expression analysis is to find stably-expressed endo-reference genes. The standard method for selection of reference genes is to evaluate the stability of candidate HK genes in RT-qPCR (Brunner et al., 2004; Czechowski et al., 2005; Basa et al., 2009; Xu et al., 2011; Pettengill et al., 2012; Wang et al., 2014). However, this method relies heavily on the candidate genes tested, which may not include genes that would have been the best internal control(s) for a given experimental system. Moreover, the standard method is unable to identify any new stably expressed genes. Theoretically, one could use high throughput expression data from microarrays or RNA sequencing to evaluate stability and then select novel HK genes. For example, using microarray data from 102 genes from legumes [all with coefficient of variation expression (CV) level <16%], Benedito et al. (2008) found many unreported stably expressed genes of unknown function along with several traditionally-used reference genes, such as glyceraldehyde-3-P-dehydrogenase and ubiquitin. Another example is in rice. Using microarray analysis, Wang et al. (2010) found 19 genes that were expressed more stably (as shown by their lower CV) than five of the commonly-used HK genes (Actin, ubiquitin, EF1α, tubulin, and DAPDH), and their expression levels were higher than all but one of the commonly-used HG genes (EF1α). In fact, the original commonly-used HK genes were not as stably expressed as expected: their expression varied dramatically among different tissues, whereas the 19 new stable expressed genes had lower CV-values. Severin et al. (2010) provide an example of selection of stable genes using RNA sequencing. Using all plant tissues, they reported that only three of the commonly-used HK genes had a CV < 20%, but if only the subset of tissues that represent seed development were analyzed, 324 HK genes had CV < 20%. In spite of brief mention of the stability of HK genes in the research described above (Benedito et al., 2008; Severin et al., 2010; Wang et al., 2010), there has been no research that specifically targeted HK genes using microarray or RNA sequencing for detection of stably expressed CE genes. Yim et al. (2015) employed 26 soybean RNA-sequencing datasets to evaluate the efficacy of reference genes reported in previous studies. They tested the specificity of reference genes through silico PCR methods, identified new and more suitable candidate reference genes through a probabilistic approach based on gaussian distribution of stability of gene expression, and further validated the expression stability of eight commonly used reference genes and seven newly identified candidates using RT-qPCR. They concluded that three of the newly identified candidate reference genes (Bic-C2, F-box protein2, and VPS-like) and one commonly used reference gene (ELF1b) gave the best overall performance under their tested experimental conditions, and that the new candidate reference genes typically exhibited more stable expression levels than many of the commonly used reference genes.

**Figure 1:**
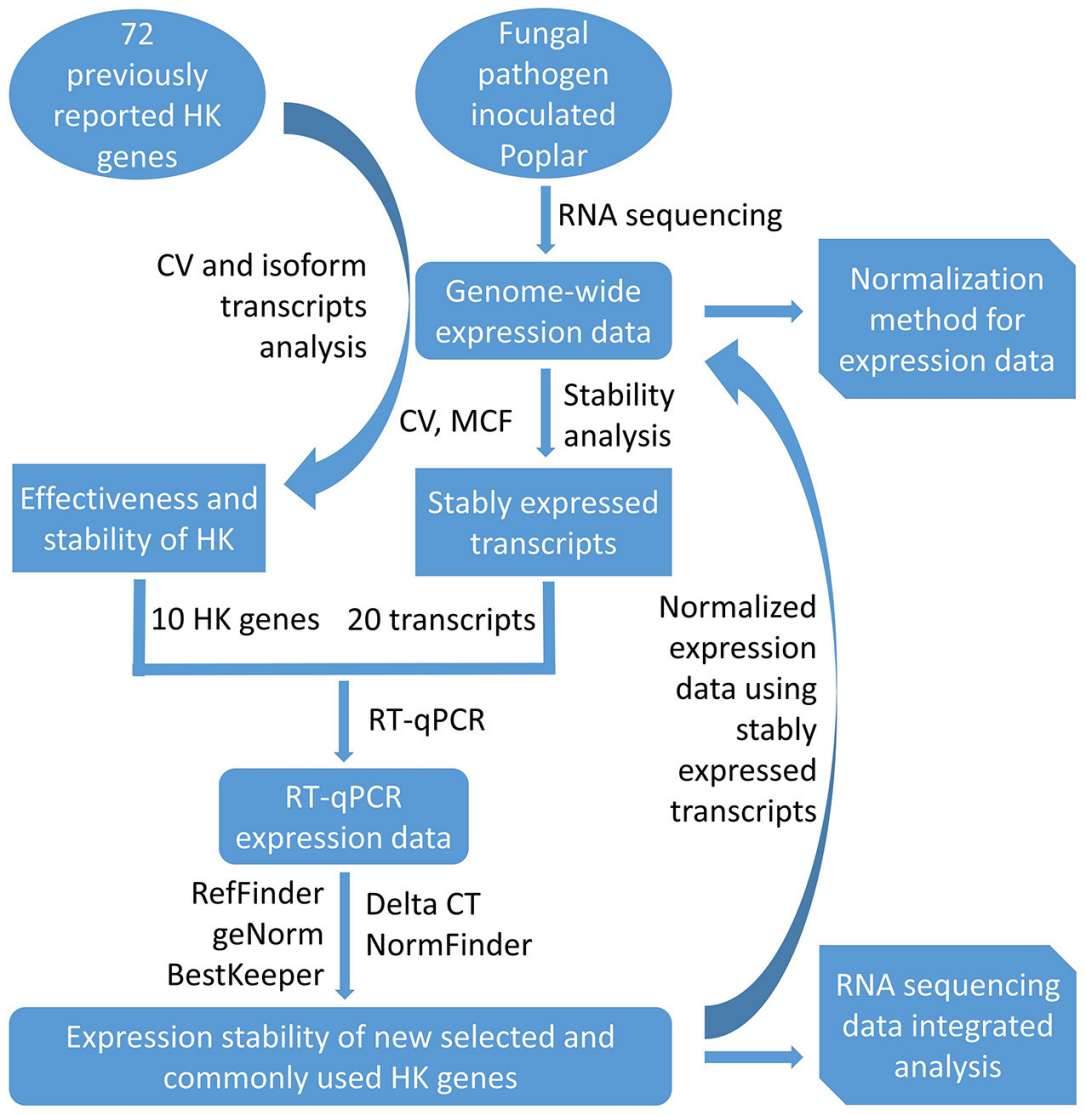
The workflow of selection of HK genes from transcript data.

This current study introduces a simple methodology for the selection of HK genes using RNA sequencing data. After first investigating the expression stability of poplar genes (including commonly used HK genes) using transcriptome analysis on xylem and phloem tissues from infected and healthy poplar stems, the study then used RT-qPCR to validate their expression stability. The potential applications of this methodology are then discussed for research that relies on normalization and/or integrative analysis of transcriptome data from different experiments, platforms, and laboratories. An overview of the workflow used here is shown in Figure 1.

## Materials and methods

### Plant and fungal materials

The study was undertaken on 6-month-old *Populus beijingenesis* clones from cuttings in the greenhouse of Chinese Academy of Forestry (CAF, Beijng, China). The pathogenic fungus used for inoculation was *Botryosphaeria dothidea*, strain CZ060, grown on 2.0% PDA medium (2.0% potato extraction, 2.0% dextrose, 1.5% agar, pH 6.0) at 25°C in the dark. Three saplings were inoculated with five mycelium discs (at 20, 30, 40, 50, and 60 cm above the soil) that were affixed over zones of stem from which a 6-mm diameter disc of the bark had been removed. A second set of three saplings were inoculated in the same way with the PDA medium but without the fungal pathogen, and are referred to as controls. Five days after inoculation, the 60 cm length of poplar stem was harvested. The buds and the five inoculation sites (0.5 cm in both directions) were removed with a razor and were discarded. To sample tissues for RNA extraction, we separated phloem from xylem, and ensured that the cambium was removed from the xylem by scraping it with a razor. There were therefore four treatment groups: disease-inoculated phloem (PI), disease inoculated xylem (XI), control phloem (PN), control xylem (XN) (*n* = 3 in each treatment group). Material was immediately frozen in liquid nitrogen and stored at −80°C until use.

### RNA extraction

Samples from each of the four treatment groups were extracted for total RNA using modified CTAB methods (Chang et al., 1993). RNA samples were characterized for purity, quantification, and integrity using agarose gel electrophoreses, the NanoDrop ND1000 spectrophotometer (NanoDrop Technologies, Wilmington, DE, USA), and the Agilent 2,100 Bioanalyzer (Santa Clara, CA, USA). A cDNA library was constructed and RT-qPCR analyses were undertaken only using the RNA samples with 260/280 ratio of 1.9–2.1 and an RIN (RNA integrity number) >7.

### Transcriptome sequencing, gene functional annotation, and gene expression analysis

The 12 RNA samples (three PI, three XI, three PN, and tree XN) were used to construct 12 cDNA libraries with fragment lengths of 200 bp (± 25 bp). Oligo dT was used to synthesize the first cDNA chain. Paired-end sequencing was then performed using the Illumina sequencing platform (HiSeq™ 2,500) according to the manufacturer's instructions (Illumina, San Diego, CA). The transcriptome sequencing was performed by Biomarker Technologies CO. LTD., Beijing, China.

Reads from each library were assembled separately. After removal of the trimming adapter sequences and the low-quality reads (those with <13 bp or with >5% of the nucleotides unknown), the clean reads were mapped to the *Populus* genome database (Phytozome10, Populus trichocarpa v3.0) (http://phytozome.jgi.doe.gov/pz/portal.html#!info?alias=Org_Ptrichocarpa) using TopHat2 (Kim et al., 2013). Functional annotation of poplar genes was performed by sequence comparison with three databases: the NCBI non-redundant protein (Nr) and non-redundant nucleotide database (Nt) (http://www.ncbi.nlm.nih.gov/), the Swiss-Prot database (http://web.expasy.org/docs/swiss-prot_guideline.html) and the Kyoto Encyclopedia of Genes and Genomes (KEGG) database (http://www.genome.jp/kegg/kegg2.html). Cufflinks (Trapnell et al., 2010) was used to evaluate transcript expression. The FPKM [Fragments Per Kilobase of exon model per Million mapped reads, defined as cDNA fragments/(Mapped fragments (millions) × Transcript length (Kb)] was used to describe the expression level of transcripts.

### Gene expression stability of poplar genes based on transcriptome sequencing data

We used the FPKM value and its logarithm as well as the count number of transcripts to estimate the level of expression of poplar genes. We used the CV (coefficient of variation) and the MFC (maximum fold change) values of the expression level to evaluate the expression stability of gene transcripts in the four environments (xylem and phloem, challenged with a pathogen vs. control). We defined a stably expressed transcript as a transcript with MFC < 2, and either a CV for the FPKM < 20% or an absolute value of the CV of logarithmically-transformed FPKM < 5%. We also summarized the gene expression levels for each of the four treatments (average and standard error).

### RT-qPCR validation of the expression stability of candidate genes

First-strand cDNA was synthesized by reverse transcribing 1 μg total RNA for each of the 12 samples using a PrimeScript II 1st Strand cDNA Synthesis Kit (Takara, Dalian, China). The RT-qPCR reactions were performed in a 25 μl volume solution containing 10 ng template cDNA, 12.5 μl 2 × SYBR® Premix Ex TaqTM II (Takara, Dalian, China) and 200 nM of each gene-specific primer. Control reactions without a template were also performed for each primer pair. The 10 μL RT-qPCR was amplified in a LightCycler® 480 (Roche Applied Science, Indianapolis, IN) under the following regime: 2 min at 50°C, 10 min at 95°C, and 45 cycles of 15 s at 95°C, and 1 min at 60°C. Two biological replicates were used for each of the 12 samples, and then six technical replicates were made for each of the biological replicates. The specificity of the amplicons was verified using melting curve analysis and agarose gel electrophoresis analysis. In total, RT-qPCR was used for validation of 20 new stably expressed transcripts and 10 commonly used poplar HK genes.

### Analysis of gene expression stability and selection of reference genes

The quantification cycle values (Cq) of RT-qPCR were used to represent the expression level of transcripts. The amplification efficiencies of tested transcripts (genes) were estimated using the LinRegPCR (http://linregpcr.nl). The web-based comprehensive tool RefFinder (http://fulxie.0fees.us/?type=reference#) (Xie et al., 2012), which integrated geNorm (Vandesompele et al., 2002), NormFinder (Andersen et al., 2004), BestKeeper (Pfaffl et al., 2004), and the comparative Delta CT method (Silver et al., 2006), were used to evaluate the stability of the HK genes.

### Comparison of reference gene analysis and cufflinks for evaluation of internal controls to normalize transcriptome data

After selecting new reference genes as described above, we tested their integrity for use as internal controls to normalize the transcriptome data. We randomly selected the transcriptome expression data of 20 genes that were differentially expressed in inoculated and non-inoculated phloem samples, calculated the expression of each gene relative to the expression level of four stable expression genes (SSU_S8e, SSU_S4e, 20S_PSU, and 26S_PRS), and then compared the expression of each gene with its expression calculated by Cufflinks. In the Cufflinks analysis, the criterion of DE gene analysis was FC > 2.0 and FDR > 0.05; for the four internal control analysis, the criterion was FC > 2.0.

## Results

### Expression profiles of commonly used plant HK genes in the poplar/canker system

First, we synthesized the biological information from previous studies on 72 commonly used plant HK genes, including gene names, transcripts names, locus in the chromosome, primer sequences, and the gene's basic or putative function (Table S1.1). Functional annotation, based on NR and NT database of NCBI, Uniprot, COG, Pfam, GO, and KEGG, revealed that these genes are mainly associated with or involved basic biological processes, cellular components or molecular functions (Table S1.2).

The FPKM values of all transcripts of these HK genes from our transcriptome data, and their expression stability (CV of FPKM, CV of logarithmic transformed FPKM and MFC) are shown in Table S2. There are 201 transcripts (Table S2) rather than 72 (Table S1.1) because alternative splicing (AS) events produced isoform transcripts of many genes. The expression data showed that 45 of the 72 commonly used HK genes had multiple transcripts (ranging from 2 to 13), including the genes for UBQ, Actin, TUA, TUB, EF1α, EIF4B-L, UBQ-L, GAPDH, and 60S RNA. UBQ, which may be the most popularly used reference gene in plants, exhibited 13 different isoform transcripts, and the Actin gene produced seven isoform transcripts. Only a minority of the HK genes (27 of the 72) had unique isoforms, and included RP-L17, EF1β, RP, TUB(3), CYP, and UBQ7 (Table S2). The FPKM data (Table S2) showed that 150 of the 201 transcripts were CE in all of the xylem and phloem samples, both diseased and un-diseased. Another 49 of the transcripts were constitutive in at least one of the 12 samples, and another 12 transcripts were not detected in any sample (Table S2).

Expression level and stability varied greatly among isoform transcripts of the same gene. Potri.001G418500, the gene that codes UBQ protein, may be the most widely-used HK gene in studies of quantitative gene expression. The average expression of its 13 isoform transcripts varied from < 1.00 to 472.26 (Potri.001G418500.1 and Potri.001G418500.7, respectively). Some of its isoform transcripts were not CE (Potri.001G418500.4), and moreover, the three transcripts with the highest expression stability (Potri.001G418500.1, Potri.001G418500.2, and Potri.001G418500.13) had much higher CVs than our adopted cut-off value of CV < 20% (40.91, 33.63, and 25.55%, respectively; Table S2). If the cut-off of CV < 30% were adopted, Potri.001G418500.13 would qualify as a stable reference gene. An issue is that the specific primers for three of the genes (UBQ(1), UBQ10-5, and UBQ11) do not distinguish among the isoforms that have the highest expression stability (Potri.001G418500.1, Potri.001G418500.2, and Potri.001G418500.13), and so the usage of UBQ as an internal control for gene expression analysis should be done with caution.

Expression level and expression stability also varied greatly among the 27 unique isoform transcript HK genes. Potri.013G101000.1 (REF), Potri.004G168800.1 (CYP), and Potri.010G150700.1 (GIIα) had high expression levels (average FPKM value 5011.54, 635.42 and 621.22) but low stability (CV of 94.71, 52.46, and 85.59%, respectively) in the poplar stem. If the less stringent cut-off value of CV < 30% were adopted, only the three most stably expressed genes (UBQ10-9, EF1β) and ACT1) would meet the criteria (CV of 15.05, 26.91, and 26.94%, respectively). Lastly, expression stability analysis showed that very few of the 72 commonly used HK genes met our threshold for stability (CV < 20%) in this experimental system (Table S2).

### Constitutive and stable expression analysis in the poplar/canker system

**Figure 2:**
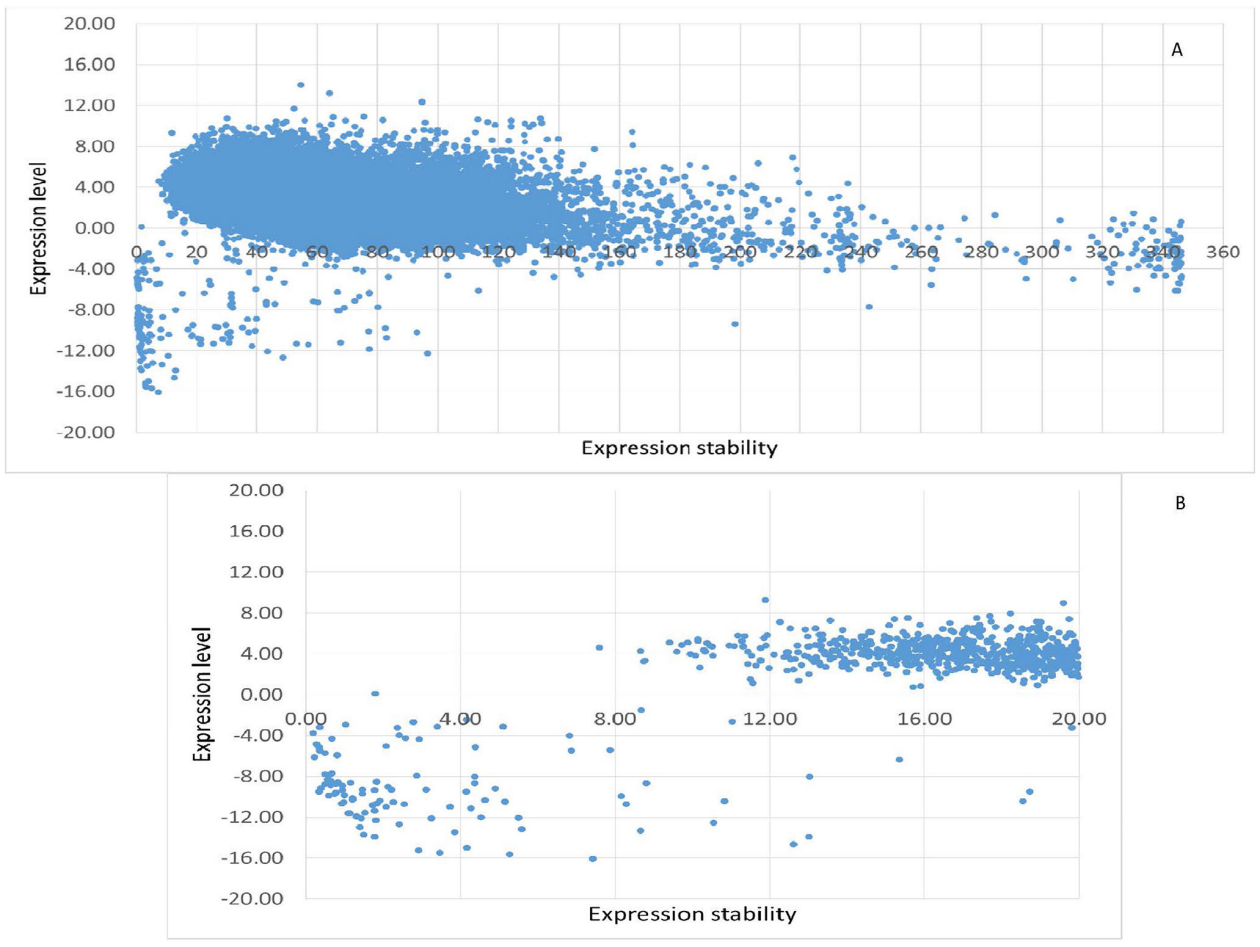
The expression characteristics of poplar constitutively expressed transcripts in the fungal/canker system. The logarithmic transformed value of FPKM is used to represent the expression level of every transcript, while the CV of FPKM is used to represent the expression stability of transcripts. **(A)** All of the constitutively expressed transcripts; **(B)** the top 729 stably expressed transcripts.

Whole genome-wide expression data (FPKM value) of 12 poplar stem xylem and phloem samples were used to evaluate the constitutive and stable expression characteristics of poplar genes. In total, 65,703 transcripts (including 290 novel identified transcripts) were derived from the transcriptome data. After removing transcripts with null expression in any of the 12 samples, there were 25,277 transcripts (of which 106 were novel) (Table S3, Figure 2A). In the other words, 38.47% of all *P. beijingenesis* transcripts were CE. After applying the criteria for the FPKM (CV < 20%) and maximum fold change (MFC < 2), 729 transcripts, or 1.11% of all poplar transcripts, were selected as the stable expressed genes from the whole-genome wide transcriptome data (Table S4.1, Figure 2B). Both FPKM and logarithmically-transformed FPKM values (for comparison to other studies) are listed in Table S4.1.

These 729 transcripts were divided into two groups on the basis of gene expression. The low expression group had 103 transcripts with an average FPKM < 1.0, which implied that their expression was low or negligible (Table S4.2). Interestingly, the expression stability was high for almost all members of the low-expression group: 93 of the 103 transcripts had CV < 8.80%.

The FPKM values of the high expression group (626 of the 729 transcripts, Table S4.3) ranged from 1.07 to 633.76 (Potri.002G059700.4, coding Ribose-phosphate pyrophosphokinase; and Potri.010G243100.8, coding DnaJ protein, respectively). Although only two previously-reported HK genes (UBQ10-9 and MSI1) were found in the high expression group (Table S4.3), functional analysis revealed that many of the transcripts in this group encoded products that were the same or related to those coded by the commonly-used HK genes, such as 21 E3 ubiquitin-protein ligase transcripts, six Eukaryotic translation initiation factor related transcripts, five actin related genes, four calcium-dependent protein kinase genes, 50S ribosomal protein and others transcripts. This analysis also provided a large pool of potential HK genes, some of which are already known to have major and important roles in plants: three 26S protease regulatory protein genes, seven 26S proteasome non-ATPase regulatory protein genes, ten serine/threonine-protein kinase genes, six serine/threonine-protein phosphatase PP2A catalytic subunit genes, ten transcription factor genes, and three 60S ribosomal export protein genes.

### RT-qPCR validation of expression stability in poplar transcripts

Based on the results of stable expression analysis, we then performed validation research using RT-qPCR. For this work we selected 20 stably expressed transcripts with CV (of FPKM) < 20% or absolute CV (of logarithmic transformed FPKM) < 5, and 10 commonly used poplar HK genes. We used agarose gel electrophoresis and melting curve-analysis to select genes with unique amplification products, and we applied the criterion of Cq < 40 to ensure that transcripts had a sufficient expression level in all conditions. The criteria for selection of reference genes were PCR amplification efficiency of the primer pairs > 1.8, and *R*^2^ of calibration curves > 0.99 (Ramakers et al., 2003). After applying these criteria, we had 10 newly selected transcripts (such as 20S_PSU, 26S_PRS, ACT_iso, ACT_uni, CDPK, DnaJ_A2, LSU_L5e, SSU_S4e, SSU_S8e, and TIF5A_iso) and nine commonly used reference genes (including 18S_RNA, CDC2, CYP, EF1β, HIS, IF5A, RA, UBQ10-5, and UBQ10-9) for the validation of expression stability (Table 1).

**Table 1:** Descriptive data related to the HK genes for which RT-qPCR was used for validation: gene name, functional description, gene, forward and reverse primer, product length, PCR efficiency, and *R*^2^ of calibration curves.

**Gene name**	**Description**	**Gene**	**Forward primer**	**Reverse primer**	**Product length**	**PCR efficiency**	***R*^2^**
SSU_S4e	Small subunit ribosomal protein S4e	Potri.015G033700	ACCCTAATAGATACAGCCTT	CGTAAAGTTGAAAAGGTCAG	121	1.931 ± 0.022	0.999
SSU_S8e	Small subunit ribosomal protein S8e	Potri.001G262100	TCACATTAAGATTGTCGTGT	ATAACGACGACTAGTTACCA	88	1.902 ± 0.019	0.998
DnaJ_A2	DnaJ homolog subfamily A member 2	Potri.008G018800	AGGAATACTCAATAACAACAA	TAAGGGAAAGAAAGAATGGAC	91	1.825 ± 0.029	0.996
CDPK	Calcium-dependent protein kinase	Potri.001G097400	CATCATATTTTGCGAGAAGTTTT	ACAATGCACACCCTAACC	80	1.928 ± 0.018	0.997
26S_PRS	26S proteasome regulatory subunit N10	Potri.009G133000	CCCAACGAATAGTTTGAATTT	CTTTGTCTAAGAGAAGACGG	86	1.945 ± 0.021	0.998
UbqCE	Ubiquitin-activating enzyme E1 C	Potri.010G031900	GTGATCAAACTTTCGAGTTC	CAGATGGCCTGAGGTTAAG	222	1.615 ± 0.065	0.993
LSU_L5e	Large subunit ribosomal protein L5e	Potri.019G099000	GTCCTTCAGATTATTTTTGCT	GTAATCCGGAATTTAATCAAG	156	1.874 ± 0.018	0.999
20S_PSU	20S proteasome subunit beta 2	Potri.017G071100	TCGCTCTAACTCTTTGAATG	CTACAAGACAGCCCTTCAT	87	1.945 ± 0.018	0.996
TIF5A_uni	Translation initiation factor 5A	Potri.008G092000	TTTGTTCTGCCATTAAGACT	AATTCTCTTCCATAGGTTCG	217	1.605 ± 0.040	0.995
TIF5A_iso	Translation initiation factor 5A	Potri.008G092000	TTTATGTGGGTTTGAGAACTGGG	CCAAGAACCACAAGAATATCATTCATT	105	1.860 ± 0.020	0.996
ACT_iso	Actin	Potri.001G309500	CCCATTGAGCACGGTATTGT	TACGACCACTGGCATACAGG	235	1.856 ± 0.026	0.997
ACT_uni	Actin	Potri.001G309500	CGACTTCAAAAGGTAAGTGA	CGACTTCAAAAGGTAAGTGA	91	1.926 ± 0.017	0.996
EF1β	Elongation factor 1-beta	Potri.001G224700	AAGAGGACAAGAAGGCAGCA	CTAACCGCCTTCTCCAACAC	145	1.885 ± 0.015	0.997
UBQ10-9	Polyubiquitin 11	Potri.011G026600	CTAAGGGTCTCTGGTTCTGCTCAA	GCTGAGACTTTTATTCAATCATTAGGAA	114	1.919 ± 0.019	0.998
UBQ10-5	Polyubiquitin 4 (UBQ4)	Potri.001G418500	GATGTGCTGTTCATGTTGTCCAA	AAGACTGCTACTGAACACACACAAGAA	90	1.923 ± 0.018	0.997
18S_RNA	18S ribosomal subunit	AY652861	TCAACTTTCGATGGTAGGATAGTG	CCGTGTCAGGATTGGGTAATTT	145	1.973 ± 0.031	0.999
CDC2	Cell division control protein 2	Potri.004G133500	TGAAACCTCAGAATTTGCTTA	TACCACAGGGTAACAACCTC	124	1.919 ± 0.019	0.996
HIS	Histone H3.3	Potri.005G072300	ACTGCTCGTAAGTCTACTGGAGG	GCGGTAACGGTGAGGCTTCTTC	111	1.897 ± 0.015	0.999
CYP	Cyclophilin	Potri.004G168800	GGCTAATTTTGCCGATGAGA	ACGTCCATCCCTTCAACAAC	174	1.923 ± 0.019	0.996
IF5A(eIF5A)	Eukaryotic translation initiation factor 5A	Potri.018G107300	TCGGACGAGGAGCACCACTT	TGCAAGGACGGTTCTTGATGACTAT	118	1.913 ± 0.013	0.997
RA	Ribulose bisphosphate carboxylase/oxygenase activase 1	Potri.008G058500	GTGGGTCTCAGGTGTTGGTGTTG	CTCTTCACATTCTCTTGCTCCTTGAC	150	1.926 ± 0.018	0.999

**Figure 3:**
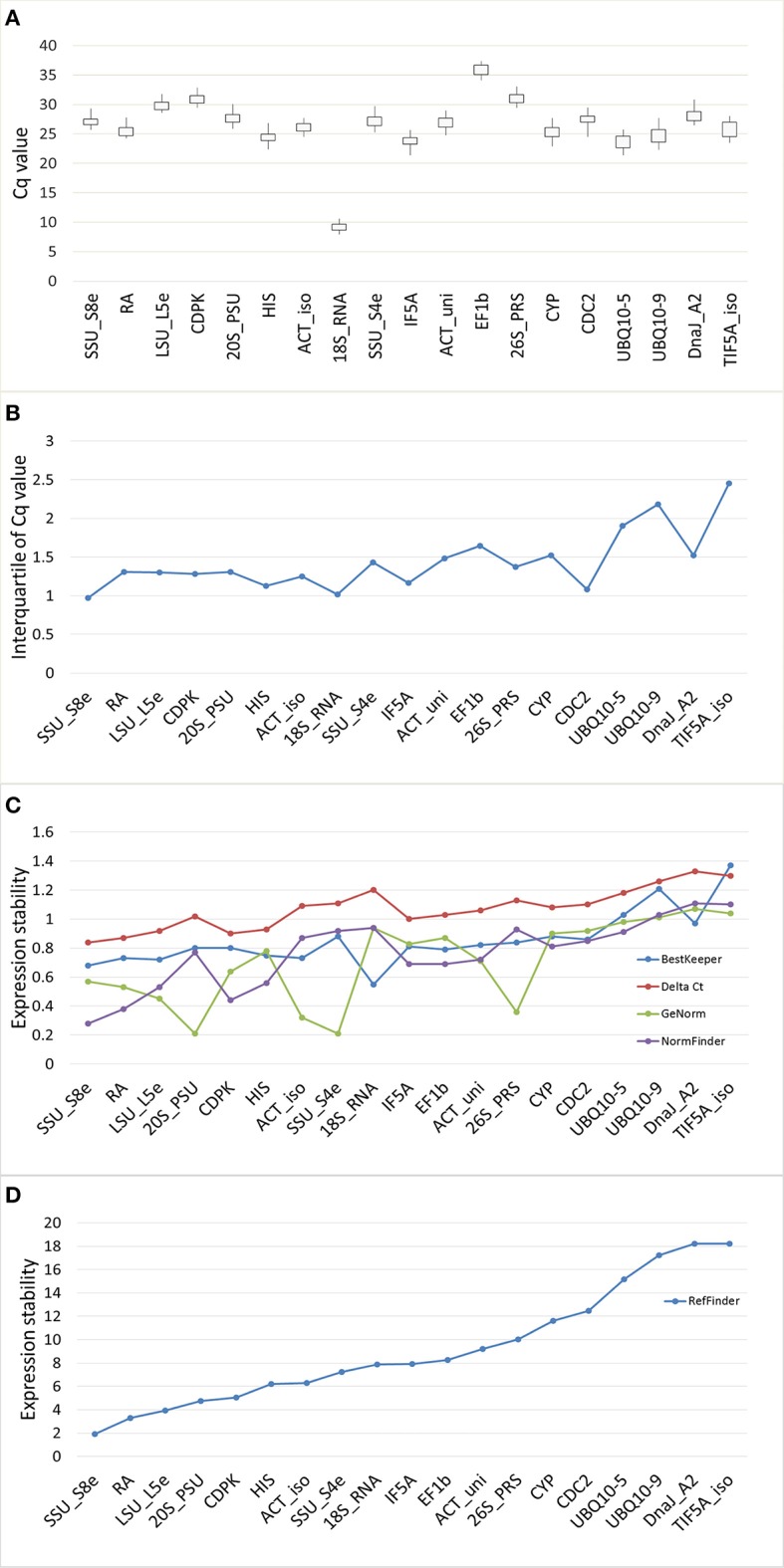
Gene expression stability of 19 candidate genes validated in RT-qPCR from the poplar/canker system. **(A)** Box-and-whisker plot showing the variation of expression of 19 reference genes. Expression data displayed as quantification cycle (Cq) values. The upper and lower points of the line represent the maximum and minimum values of Cq, and the upper and lower lines of the box represent the 25th percentile (P25) and 75th percentile (P75). **(B)** The interquartile analysis showing the stability of candidate genes. **(C)** Comparison the ranking of expression stability of 19 candidate genes in GeNorm, Best Keeper, Delta CT, NormFinder. **(D)** The comprehensive ranking of candidate genes in RefFinder.

There was large variation in raw expression levels among the 19 reference genes, 18S_RNA showed the highest expression with an average Cq of 9.09; EF1β showed the lowest expression with an average Cq of 35.80 (Figure 3A, Table S5). The expression level of 18S_RNA was almost one thousand times greater than the level of the second highest gene (UBQ10-5, average Cq of 23.57), while the expression of UBQ10-5 was one thousand times that of EF1β. Variation of the expression data was evaluated using the standard error (STDEV) and the interquartile value. The transcript for SSU_S8e had the lowest STDEV (0.68) and the lowest interquartile values (0.68 and 0.97, respectively), and TIF5A_iso had the highest values (1.37 and 2.45, STDEV and interquartile values, respectively). Based on STDEV and interquartile analysis, SSU_S8e showed the most stability and TIF5A_iso showed the least in this fungal inoculated poplar system (Figures 3B,C, Table S5).

We also evaluated the stability of the 19 candidate reference genes through the comprehensive online tool RefFinder. RefFinder calculates expression stability of each gene in five ways: using its own algorithm and using four commonly-used programs, geNorm, NormFinder, BestKeeper, and Comparative Delta Ct. In geNorm program, the average expression stability for each gene (M) was calculated based on the average pairwise variation between all tested genes. The most stably expressed genes identified by geNorm were 20S_PSU and SSU_S4e (*M* = 0.21); the least stably expressed gene was DnaJ_A2 (*M* = 1.37). When *M* < 1.5 (the cut-off value for internal control selection recommended by geNorm) was adopted, even the least stable gene, DnaJ_A2, qualified for use as an internal control in RT-qPCR. However, when a rigorous criterion (*M* < 0.5) was adopted, only five genes (20S_PSU, SSU_S4e, LSU_L5e, 26S_PRS, and ACT_iso) qualified (Table 2, Figure 3C). Moreover, the stability ranking of 19 candidate HK genes was showed in Table 2 and Figure 3D.

**Table 2:** Expression stability and its ranking of 19 candidate HK genes evaluated by five methods.

**Genes**	**BestKeeper**		**Delta Ct**		**GeNorm**		**NormFinder**		**RefFinder**	
	**STDEV [±CP]**	**Ranks**	**Average of STDEV**	**Ranks**	**Stability value**	**Ranks**	**Stability value**	**Ranks**	**Geo-mean of ranking values**	**Ranks**
SSU_S8e	0.68	2	0.84	1	0.57	7	0.28	1	1.93	1
RA	0.73	4	0.87	2	0.53	6	0.38	2	3.31	2
LSU_L5e	0.72	3	0.92	4	0.45	5	0.53	4	3.94	3
20S_PSU	0.8	8	1.02	7	0.21	1	0.77	9	4.74	4
CDPK	0.8	8	0.9	3	0.64	8	0.44	3	5.05	5
HIS	0.75	6	0.93	5	0.78	10	0.56	5	6.22	6
ACT_iso	0.73	4	1.09	11	0.32	3	0.87	12	6.31	7
SSU_S4e	0.88	14	1.11	13	0.21	1	0.92	14	7.23	8
18S_RNA	0.55	1	1.2	16	0.94	15	0.94	16	7.87	9
IF5A	0.81	10	1	6	0.83	11	0.69	6	7.93	10
EF1β	0.79	7	1.03	8	0.87	12	0.69	6	8.28	11
ACT_uni	0.82	11	1.06	9	0.71	9	0.72	8	9.19	12
26S_PRS	0.84	12	1.13	14	0.36	4	0.93	15	10.02	13
CYP	0.88	14	1.08	10	0.9	13	0.81	10	11.61	14
CDC2	0.86	13	1.1	12	0.92	14	0.85	11	12.45	15
UBQ10-5	1.03	17	1.18	15	0.98	16	0.91	13	15.18	16
UBQ10-9	1.21	18	1.26	17	1.01	17	1.03	17	17.24	17
DnaJ_A2	0.97	16	1.33	19	1.07	19	1.11	19	18.2	18
TIF5A_iso	1.37	19	1.3	18	1.04	18	1.1	18	18.24	19

**Figure 4:**
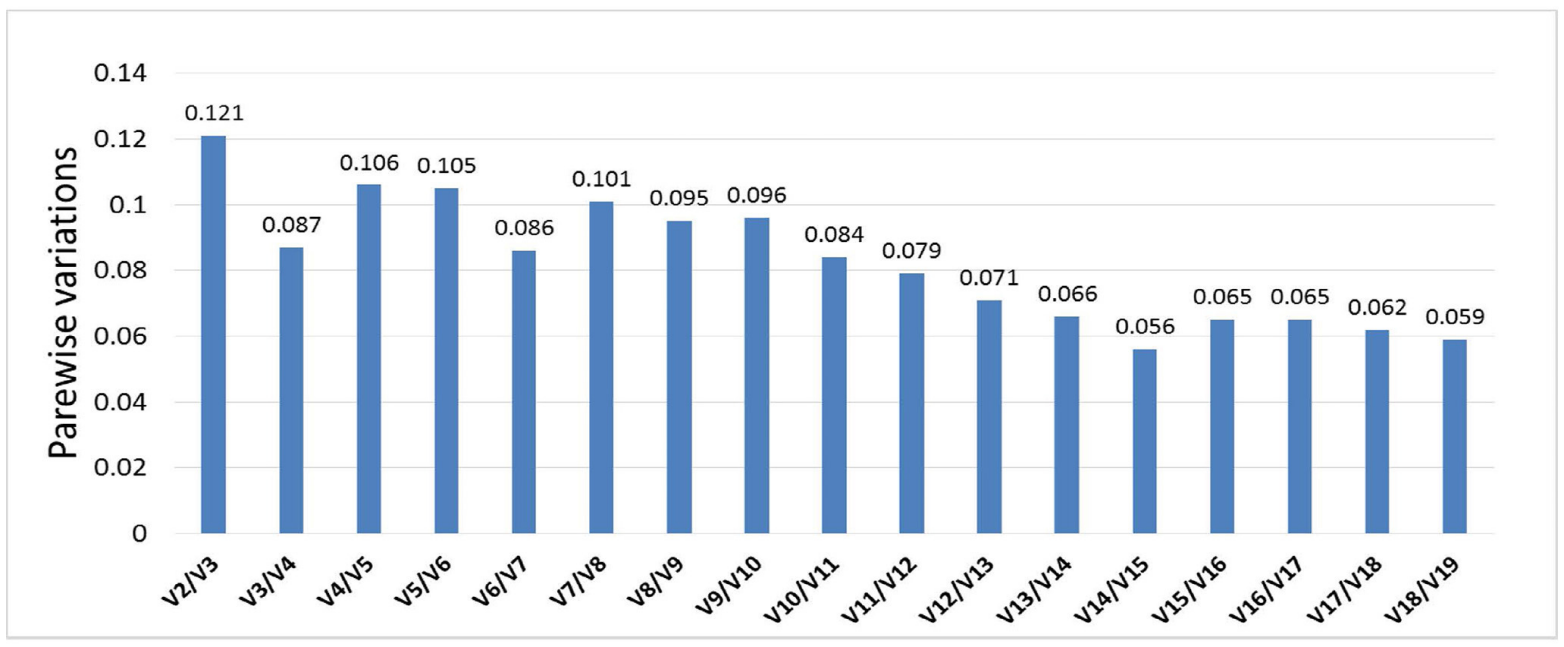
Pairwise variation analyses determined the optimal number of control genes for normalization. Results showed that all pairwise variations of 19 genes were under the cut-off value 0.15, then the top 2 stably expressed genes should be used in data normalization in RT-qPCR.

Optimal number of reference genes was calculated using pairwise variation (Vn/Vn + 1) in geNorm (Vandesompele et al., 2002), using a cut-off value for the optimal number of reference genes at 0.15. This research suggests that SSU_S8e and RA, the two genes with the highest stability of the 19 tested (Table 2, Figure 4), are the best internal controls for expression data normalization.

### Comparison of reference gene analysis and cufflinks for evaluation of internal controls to normalize transcriptome data

For 16 of the DE 20 genes, the five data normalization methods yielded identical results regarding the direction of gene expression relative to controls (e.g., whether up- or down-regulated) and also gave quite similar estimates of the magnitude of the relative gene expression (**Figure 6**). For three of the 20 genes, the five data normalization methods showed significant DE in inoculated vs. non-inoculated samples, with much higher values of relative gene expression from the four internal control analyses than from the Cufflinks analysis. Finally, for transcripts Potri.009G017600.1, none of the four internal control analyses revealed the DE of its transcript as revealed by Cufflinks. When we further investigated the expression data of these last four transcripts, we found that the differences mainly came from either the trace expression of transcripts or the uneven expression level among three biological replicates.

## Discussion

### Constitutive expression analysis and mining the stable expressed genes from transcriptome data

Most transcriptome analyses are mainly focused on expression and function of differentially expressed (DE) genes, with relatively fewer studies (Benedito et al., 2008; Severin et al., 2010; Wang et al., 2010) on the characteristics of CE and stably expressed genes. The present study focused on these constitutively and stably expressed genes. Our CV and MFC analysis revealed that 38.47% of all *Populus beijingenesis* transcripts were CE, and 1.11% of all poplar transcripts were expressed stably for the four cases studied (poplar xylem and phloem, with and without fungal pathogen inoculation).

### Potential application of new stably expressed genes in gene expression normalization

Ribosome is the factory of protein synthesis in all cellular organisms. All ribosomes are composed of two subunits, both of which are built from RNA and protein. This study showed through both transcriptome analysis and RT-qPCR that four of the genes coding for ribosomes are stably expressed in poplar branches: SSU_S8e and SSU_S4e, which code for the small subunit S8e and S4e proteins, respectively; 18S_RNA, which codes for the ribosomal gene 18S rRNA; and LSU_L5e, which codes for the large subunit L5e protein.

Gene SSU_S8e and gene LSU_L5e were the most- stable and third-most stably expressed genes in the comprehenisve RefFinder analysis, and SSU_S4e was the most stably expressed gene in GeNorm analysis. The 18S_RNA gene is a multiple-copy gene, with 4,000 copies of rDNA units in the genomes of *P. nigra, P. deltoides*, and *P. maximowiczii* (Faivre-Rampant et al., 1992). The current RT-qPCR analysis revealed that in *P. beijingensis* from four contexts, the 18S_RNA gene had both high expression and high stability (average Cq value 9.09), which was thousands of times higher than other candidate genes tested (average Cq-value from 23.57 to 35.80). Because of their stability with a fungal pathogen in both xylem and phloem tissues, this research shows that these four ribosome-related transcripts should be taken as new HK genes for gene quantification. 18S_RNA will find use in data standardization of the genes with high expression levels [such as Ribulose-1,5-bisphosphate carboxylase/oxygenase, or Potri.013G100800 (phosphorylase superfamily protein coding gene, the highest expressed gene in the fungal pathogen inoculated poplar)]. The other three genes will find use in analysis of induced expression genes.

A plant's ability to alter growth and development relies heavily on its proteomic plasticity. Plants use a variety of methods to control the level and activity of their constituent proteins. The Ubiqutitin/26S proteasome (Ub/26S) system is one of the most important proteolytic pathways in eukaryotes, whereas the 20S proteasome is a key element of the Ub/26S system (Hochstrasser, 1995; Groll and Huber, 2003).

This study showed that the gene 26S_PRS (encoding the 26S proteasome regulatory subunit N10) and the gene 20S_PSU (encoding the 20S proteasome subunit beta two protein) had higher stability (by ranking) than did two commonly used internal control poly-ubiquitin genes (UBQ10-5, UBQ10-9). These results are suggestive that these two genes are good candidate HK genes for studies in the proteasome system. The Ub/26S proteasome pathway is involved with one of the most elaborate regulatory mechanisms in plants; a near-complete study of the *Arabidopsis thaliana* genome identified more than 1,300 genes (5% of the genome) involved in the Ub/26S system (Vierstra, 2003). Our research found 37 stably expressed transcripts involved in this system (10 26S proteasome related transcripts, 21 E3 ubiquitin protein ligase transcripts, 4 ubiquitin-conjugating enzyme transcripts and two polyubiquitins), giving further support to the use of the Ub/26S system as a rich source for HK gene selection.

### Selection of new HK genes from transcriptome data

The literature shows a wide variety of ways to find new HK genes, and the current study evaluated the efficacy of several of these methods. Typically, for a transcriptome analysis, FPKM and/or RPKM (Reads Per Kilobase of exon model per Million mapped reads) are used to measure the expression level of transcripts. In some studies, the count number of transcripts is also used. MFC, STDEV, and interquartile of expression level are the most commonly-used metrics to evaluate the variation among the data, with CV as the most popular parameter to evaluate expression stability of genes (Benedito et al., 2008; Severin et al., 2010; Wang et al., 2010; Daines et al., 2011; Graveley et al., 2011; Yin et al., 2015). Typically, the top 1,000 expressed transcripts with the lowest CV values in contrasting environments are taken as the stably expressed genes (Zhu et al., 2008; Van Hiel et al., 2009; Severin et al., 2010; Yin et al., 2015), with cut-off threshold values that are typically CV < 16 or < 20% (Benedito et al., 2008; Severin et al., 2010; Wang et al., 2010). An alternative method for selection of stably expressed genes is to choose genes whose RNA-seq has a sufficiently low CV of its logarithmically transformed RPKM and logarithmically transformed transcript copy numbers (threshold typically CV < 4%) (Fernández-Aparicio et al., 2013).

**Figure 5:**
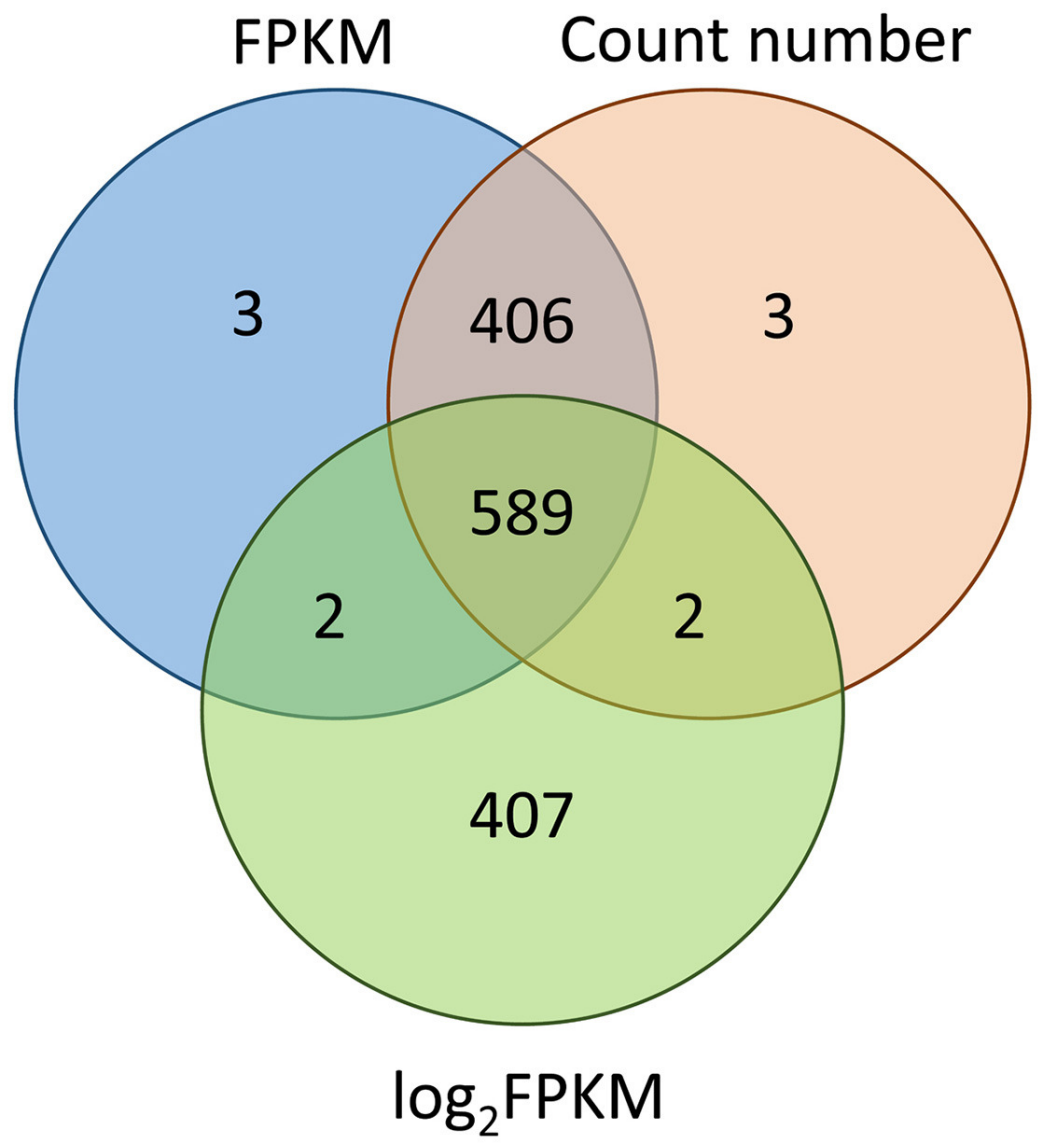
This consistency analysis among the output of CV of FPKM, CV of count number of reads, and CV of logarithmic-transformed FPKM. The 1,000 most stable transcripts derived from these three methods respectively. Results showed that FPKM (non-transformed) and count number methods gave almost the same results (sharing 99.7% of the 1,000 transcripts), while the FPKM (or count number) method and the logarithmic transformed FKPM method shared only 59.7% of 1,000 transcripts.

In this study, we evaluated the consistency of the outputs from three different methods: CV of FPKM, CV of reads count number and CV of logarithmically transformed FPKM. Results revealed that the FPKM method gave almost the same results as the count number method, sharing 99.7% of the expressed transcripts. In contrast, the FPKM method (or count number method) and the logarithmic transformed FKPM method shared only 59.7% of 1,000 transcripts (Figure 5). We also evaluated whether the cut-off threshold of 20% for the CV of FPKM was appropriate: SSU_S8e had with CV value 23.46% and logarithmic transformed CV value 3.91%, SSU_S4e had with CV value 24.07% and logarithmic transformed CV value 4.00%. RT-qPCR analysis illustrated that both of them were quite stably expressed in poplar, implying that the standard practice of CV can be relaxed and that CV (of FPKM) < 30% can be used as the cut-off threshold value for stably expressed gene selection.

The expression level of transcripts is another factor that should be considered when selecting reference gene from transcripts data. Theoretically, transcripts with both low and high expression from Table S4 are potential internal controls for gene quantification, but for reasons of efficiency and accuracy, those transcripts with the higher expression level are superior choices. In a rice genome microarray analysis, expression unit of 100 was taken as the minimum threshold level in HK gene selection (Benedito et al., 2008). In our RT-qPCR validation experiment, the average FPKM value of the tested transcripts (genes) varied from 26.99 to 480.24, and these transcripts (genes) all reached the quantification cycle (Cq) at values < 40 in RT-qPCR. This result suggested that the cDNA template of this experiment was at an appropriate level. On this basis, we recommended adoption of an FPKM value of 50 to 1,000 as the cut-off threshold value for HK gene selection from transcriptome data.

Alternative splicing (AS) is one of the most important mechanisms for generating a large number of mRNA and protein isoforms from a relatively small number of genes. AS events change the structure of transcripts and their encoded proteins, and thus provide a level of functional plasticity for eukaryotic organisms. With genome data, one can easily deduce the number of AS events or the number of isoform transcripts (http://phytozome.jgi.doe.gov/pz/portal.html#!info?alias=Org_Ptrichocarpa). In *P. trichocarpa*, 27,005 genes had one unique form transcript; these forms accounted for 37.02% of all the transcripts. The remaining transcripts came from 14,251 genes that had from 2 to 23 isoform transcripts. In the current research, about two third of the *P. beijingensis* transcripts had two or more isoform transcripts, including not only the commonly used HK genes (54 of 72 genes) (Table S2) but also the 230 genes (out of 729) with the highest expression stability (Table S4).

For these HK genes and high-stability expression genes, our results also revealed that the expression level was extremely variable among the isoform transcripts; in some cases they were not even detectable in all samples (Tables S2, S4). Additionally, the sequence alignment analysis revealed that many of the reported primers of HK are not specific to certain isoform transcripts (such as UBQ11, UBC5); instead, the amplification products of these primers are a mixture of isoform transcripts. This fact leads to the possibility that the expression could be inaccurate when normalized by these internal controls.

Therefore, we designed two different primer pairs for each of two genes (ACT and TIF5A), one primer pair that could amplify a specific transcript and the second primer pair that could amplify two or more isoform transcripts in one tube. Three of these primers are reported in this study: ACT_iso for seven isoform transcripts of Potri.001G309500, ACT_uni for Potri.001G309500.7, and TIF5A_iso for two isoform transcripts of Potri.008G092000. The fourth primer (TIF5A_uni) is not reported here; it was removed due to nonspecific amplification. The comprehensive ranking revealed that the expression stability resulting from the primer ACT_iso was higher than from the primer ACT_uni, but the experimental design did not allow us to test the significance of this difference. Nonetheless, we suggest that the most secure strategy for selection of stable expression genes is to use those genes with a unique product from their gene transcription.

Based on this research, we therefore make the following recommendations for selection of HK genes from transcriptome data: chose genes that have (1) a unique product from their transcription, (2) constitutive expression in all samples, (3) a CV value of expression < 20% (or possibly 30%) and an MFC value of expression level < 2, and (4) an expression level from 100 to 1,000 units.

### Use of stably expressed genes in normalization of transcriptome data

**Figure 6:**
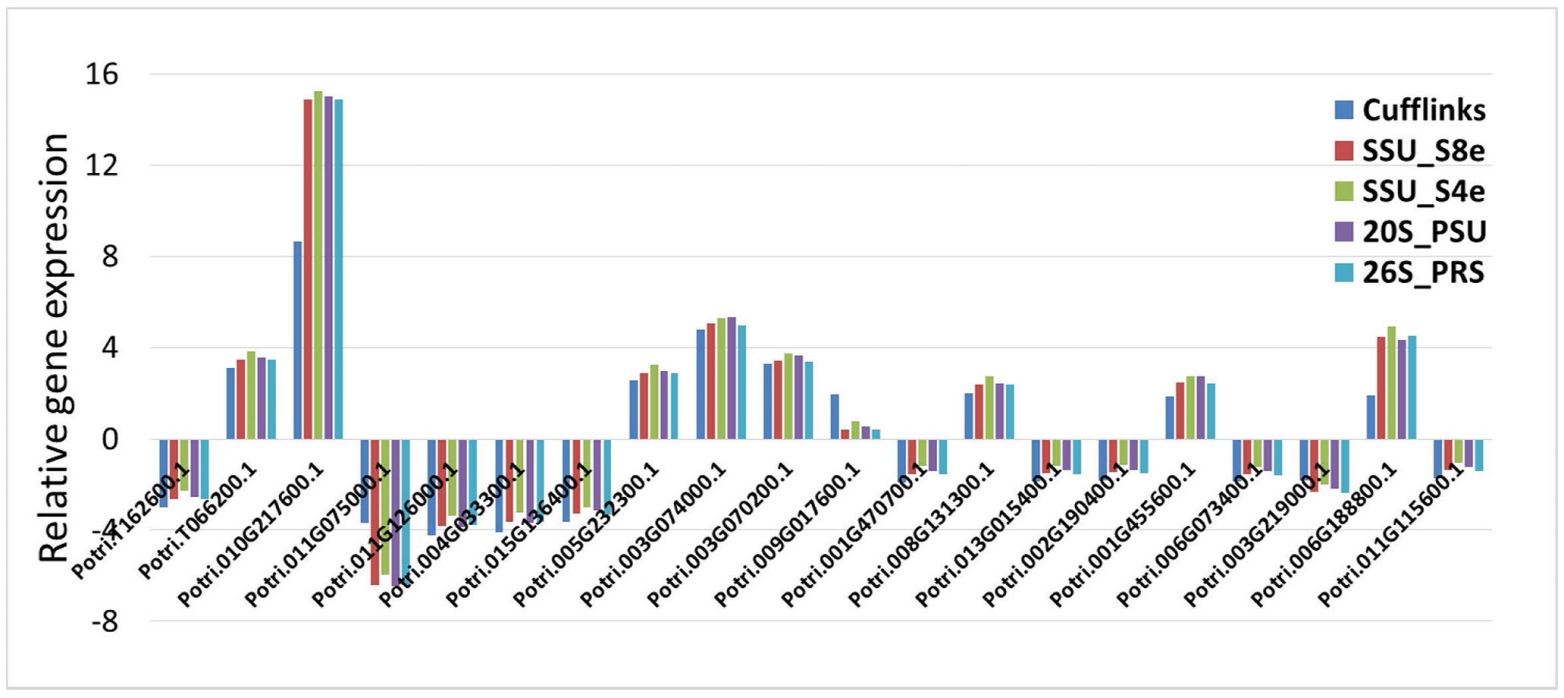
Evaluation the potential of HK genes used in transcriptome data normalization and differential expressed analysis. The relative expression of 20 differential expressed genes from phloem samples were reevaluated based on four HK genes (SSU_S8e, SSU_S4e, 26S_PRS, and 20S_PSU) normalized FPKM data, then compared the outputs with that of Cufflinks analysis.

RNA sequencing has rapidly become an invaluable tool to characterize transcriptomes. Together with the growing usage of RNA sequencing, a number of data analysis methods and pipelines have been developed for data normalization and DE between distinct samples (sample groups). Some researchers have made comprehensive comparisons of the normalization methods or software packages for the differential analysis of Illumina high-throughput RNA Sequencing data (Dillies et al., 2013; Li et al., 2015; Seyednasrollah et al., 2015; Lin et al., 2016). For simulated data, TC, UQ, Med, Q, and RPKM normalization methods have not been able to control false positives in data having a small number of genes with very high read counts, while the DESeq and TMM normalization methods are designed to account for these extreme differences in read count number (Dillies et al., 2013). Spearman correlation analysis revealed that RC, UQ, Med, TMM, DESeq, and Q did not noticeably improve gene expression normalization, regardless of read length. Sailfish with RPKM gave the best normalization results when alignment accuracy was low; RC was sufficient for gene expression calculation when alignment accuracy was high (Li et al., 2015). A last question addressed by the current research, is whether newly-identified stably expressed HK genes can be used as internal controls in the normalization of RNA sequencing data. As shown in Figure 6, 19 of the 20 genes identified differentially expressed characteristics in our four reference gene normalization analyses, which were very similar to the results in Cufflinks analysis. Therefore, the use of these stable expression genes may provide a fast, easily-manipulated, and empirically-simple method to carry out DE analysis using transcriptome expression data.

As sequencing data continue to accumulate, the challenge emerges of how to identify differentially expressed genes among various RNA-seq datasets obtained from different experimental designs, laboratories, and platforms. One study compared the outcomes of seven sequencing centers for the sequencing in one large system (mRNAs and small RNAs of lymphoblastoid cell lines of 465 individuals) ('t Hoen et al., 2013). They concluded that given proper standardization and randomization procedures, it was feasible to distribute RNA sequencing among different laboratories. Using the methodology presented here, it would be possible to carry out an integrating analysis among experiments if the same genes were stably expressed in different databases. The exact genes identified in the current study may not be suitable for normalization in other datasets, given that their expression patterns would change over time, and they derived from only one time point (5 days-after-inoculation). However, one piece of evidence suggests this method may have some measure of universality: two of the genes that were shown here by RT-qPCR to be stably expressed (EF1β and CDPK) were also identified in another dataset from an experiment not reported here, carried out in a different poplar species and infected by two, not one, fungal pathogen. In coming years, there will be an increasing quantity of gene expression data from different protocols, environments, sequencing platforms, and even species. This methodology may have a role in helping integrate and mine such data in a meaningful way.

## Conclusion

Based on the expression stability analysis and RT-qPCR validation, we demonstrated a new methodology to select the stably expressed genes from transcriptome expression data. In comparison with the traditional HK genes selection method, our method is more targeted, convenient, and efficient for finding new and more stable expression genes. These stable genes, selected from transcriptome data, can then be used with simpler protocols to carry out DE gene analysis on transcriptome data. These genes also can be conveniently and reliably used in RT-qPCR analysis in the current experiments. Actually, we believe this method should be taking as a necessary step in gene expression analysis, especially in this genome era. Moreover, this methodology provides a new pathway that may find use in the integration and deep mining of the emerging abundance of gene expression data from different environments, laboratories, sequencing platforms, even species.

## Author contributions

JZ conceived and designed the experiments; JZ and JF analyzed the data; JZ wrote the paper; JZ, BL, and XW revised the paper; FY, YW, and JW carried out plant inoculation and real time qPCR experiments; all authors reviewed the paper.

### Conflict of interest statement

The authors declare that the research was conducted in the absence of any commercial or financial relationships that could be construed as a potential conflict of interest.
